# Indole microbial metabolites: expanding and translating target(s)

**DOI:** 10.18632/oncotarget.19443

**Published:** 2017-07-22

**Authors:** Sridhar Mani

**Affiliations:** Departments of Medicine and Genetics, The Albert Einstein College of Medicine, Bronx, NY, USA

**Keywords:** indole, nuclear receptor, inflammation, innate immunity, cancer

A central thesis of mechanisms of action of tryptophan and its metabolites in the intestines, have broadly implicated nuclear receptors and other host enzymes. The notion that all indole metabolites, of necessity, have the same degree and predictable interactions with nuclear receptors has clearly been challenged. The existence of receptors that complement actions of other well-known receptors has now taken center-stage in elucidating the roles of tryptophan metabolites in the host. Our laboratory, along with other laboratories working in this space, has sought to explore these interactions in depth. This commentary serves as a summary of ongoing work on the interactions of indole metabolites, specifically those produced by microbiota and nuclear receptors in eukaryotes.

Recent work on indoles, specifically those generated via microbial metabolism, show that several host receptors might be salient targets regulating host-microbe homeostasis. Some include the aryl hydrocarbon receptor (AhR) [1], the pregane X receptor (PXR) [2], dual effects on GLP1 release via a stimulatory action mediated by voltage-gated K^+^ channel blockade and an inhibitory effect caused by suppression of mitochondrial metabolism [4], and free-radical scavenging. It is likely that the eventual interplay between receptors and metabolites will be decided by the relative effects each of these and other receptor systems to be discovered, will have, under conditions where metabolite concentrations change. A prime example of this is seen with short chain fatty acids (SCFA) - there is not one dominant system of receptors that govern all aspects of SCFA effects *in vivo* [3–5]. However, it is crucial that we dissect each mechanism, no matter how trivial, as these data will substantively inform a systems approach in the future.

In keeping with this idea, our laboratory has embarked on using the Pregnane X Receptor (PXR) as a prime target for indole propionates and like analogs. The importance of PXR (and AhR) in regulating intestinal innate immunity, inflammation and perhaps even colitis-induced colon cancer has now been well demonstrated in the literature. Based on our studies, we hypothesize that the synergistic binding of indole(s) to the human PXR ligand binding domain (LBD) can be chemically mimicked (bacterial metabolite mimicry) leading to the discovery of novel, more potent chemical entities and drugs that activate PXR and repress inflammation [6]. Thus, in our future studies we will develop and test endobiotic PXR ligand analogs to modulate the activation of the receptor as a targeted approach for treating intestinal inflammation. Specifically, we have developed a strategy to screen and test for novel indole mimics of IPA that have the unique potential to selectively activate PXR yet preserve desired off-target properties. Conceptually, these endobiotic mimics are a departure from the existing PXR ligands. Leads developed and chosen from our cell line screens will advance into initial testing in humanized and PXR^−/−^ mice. Indeed, our group has generated floxed mice (targeting exon 5 of the PXR gene using constructs obtained from the European Conditional Mouse Mutagenesis Program (EUCOMM)), which will then allow us to generate tissue-specific PXR knockout mice. We have extensive experience with use of these models in further developing chemical and genetic models of colitis.

An important aspect of developing small molecules is to define the biochemistry of indole interactions with PXR. In collaboration with The Freire Laboratory at Johns Hopkins, we have used isothermal titration calorimetry and isothermal chemical denaturation to investigate indole interactions with PXR protein in solution. These studies will provide us with critical binding affinity estimates to pure protein in solution and help guide compound selection. A full complement of chemical analogs have been developed that mimic the indole propionate pharmacophore and these studies are ongoing.

Overall, our commentary provides a synopsis of the work underway that will eventually define potent and hopefully non-toxic modulators of PXR (as well as PXR/AhR) in the intestines. There are several hurdles that need to be addressed; however, with the expertise available we hope to position this aspect of drug mining as a feasible means towards finding clinically applicable drugs for human inflammatory and other barrier disrupting diseases.
